# The influence of denture adhesives on the microhardness, color, and roughness of rigid reliners, before and after thermocycling

**DOI:** 10.2340/biid.v12.45226

**Published:** 2025-12-29

**Authors:** Bárbara Luise Medeiros dos Santos, Daniela Micheline dos Santos, Gabriele Martins, Fernanda Pereira de Caxias, Beatriz Miwa Barros Nakano, Marcelo Coelho Goiato

**Affiliations:** Department of Dental Materials and Prosthodontics, Araçatuba School of Dentistry, São Paulo State University (UNESP), São Paulo, Brazil

**Keywords:** Reline, adhesives, microhardness, roughness, color

## Abstract

The stability and functionality of complete dentures depend on various clinical and material factors, such as retention, adaptation, color change, microhardness, and surface roughness. Relining aims to re-establish the fit of the prosthesis to the supporting tissues, and it is carried out using materials such as acrylic resins. This study aimed to evaluate the color, microhardness and roughness properties of three relining acrylic resins: Kooliner (GC America, USA), TDV-Cold (TDV, Brazil), and Ufi Gel Hard C (VOCO, Germany), after combined use with three different adhesives (Corega, Fixodent – Haleon, UK; and Poligrip – P&G, USA) for 2 months, followed by aging by thermocycling (5.000 cycles). Color, microhardness, and roughness were analyzed on 120 samples at times T0 (initial), T1 (after 2 months), and T2 (after thermocycling). Additionally, characterization by energy-dispersive X-ray spectroscopy and scanning electron microscopy was performed at T0 and T2. The results showed that time had a significant influence on all the properties evaluated, with an increase in microhardness and roughness over time. Ufi Gel Hard C showed greater color stability and microhardness, even after aging, a behavior associated with the presence of elements such as silicon and barium in its composition. Roughness was also more evident in this relining acrylic resin, as confirmed by the SEM data and images. The reliners analyzed showed distinct behavior in response to aging and thermocycling, with Ufi Gel Hard C standing out for its greater stability. Time was the main factor influencing color, microhardness, and roughness properties.

## Introduction

Prosthodontics is a science aimed at replacing missing teeth to restore the patient’s chewing functions, aesthetics, comfort, and oral health. There are different types of dentures, such as complete dentures, which replace all the teeth in an arch, partial dentures, and single dentures. Conventional dentures made from digital scans (DSs) for completely edentulous arches remain a challenge [1, 2].

Relining complete dentures consists of adjusting the internal surface of the denture to improve the fit of the prosthetic base to the supporting tissues, which change over time due to continuous bone resorption. This procedure is essential for restoring the comfort, stability, seating, and retention of the prosthesis, factors which are often compromised by the loss of bone support [3, 4]. Relining materials, such as acrylic resins and silicones, are used to coat all or part of the denture surface that directly contacts the oral mucosa. In addition to promoting better adaptation, these materials also help to distribute masticatory forces more evenly and absorb the energy generated during function, contributing significantly to the clinical performance of the prosthesis [4, 5].

Regarding properties, color stability, surface hardness, and smoothness are crucial characteristics for the durability of prostheses. Color change can indicate aging or damage to materials [6], surface hardness affects abrasion resistance [7], while surface smoothness promotes comfort and aesthetics, reducing the accumulation of bacteria [8]. The thermocycling, a procedure used to simulate how materials would age in a real clinical situation, promotes successive volumetric contractions and expansions of the materials, causing their degradation [8]. This degradation can be reflected in the discoloration of the base of acrylic resins used in complete dentures and a reduction in their durability [9].

The effect of rigid relining materials on the color change, microhardness, and roughness of acrylic resin-based prostheses is an aspect that has been little explored in the scientific literature to date. Thus, this study aimed to evaluate the color, microhardness, and roughness properties of three relining acrylic resins: Kooliner (GC America, Alsip, USA), TDV-Cold (TDV, Pomedore, Brazil), and Ufi Gel Hard C (VOCO, Cuxhaven, Germany), after combined use with three different adhesives (Corega, Fixodent – Haleon, Brentford, UK; and Poligrip – P&G, Cincinnati, USA) for 2 months, followed by aging by thermocycling. Energy dispersive X-ray spectroscopy (EDS) analysis, and scanning electron microscopy (SEM) were also carried out to complement the results and discussion.

The null hypothesis was that the adhesives would not influence color change, microhardness, and roughness of the relining acrylic resins before and after aging by thermocycling.

## Materials and methods

### Study design

**Figure 1:**
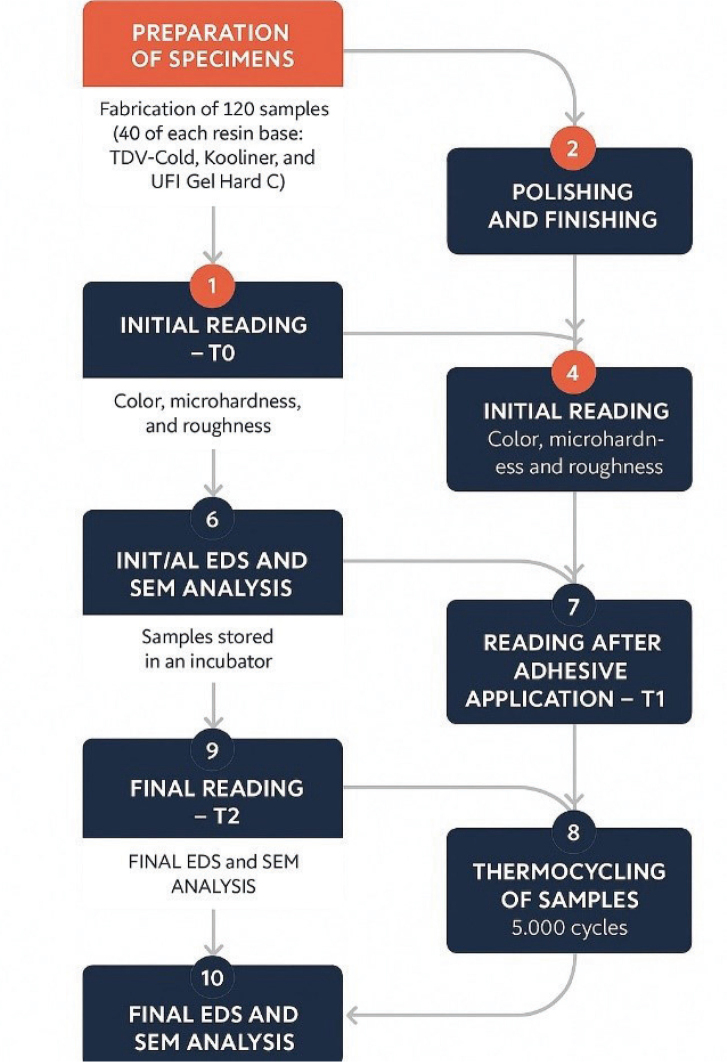
Schematic representation of the methodological stages of the study, from sample preparation to final analysis of the parameters evaluated.

A total of 120 samples were made, of which 40 were fabricated using the acrylic resin-based reliners Kooliner GC, 40 were fabricated using TDV-Cold, and the other 40 were fabricated using the Ufi Gel Hard C. The samples were submitted to analysis of color change, microhardness, and roughness at T0 (initial). Then, the samples were separated into groups: 10 were the control samples, which did not receive adhesives for removable prostheses, 10 samples received Fixodent adhesive, 10 samples received Poligrip adhesive, and 10 samples received Corega adhesive. Table 1 represents the materials used for the fabrication. The samples were again submitted to analysis of color change, microhardness, and roughness at T1 (after 2 months), and T2 (after thermocycling). The samples were also analyzed by EDS and SEM at T0 and T2. Figure 1 represents the sequence of the study.

**Table 1:** Description of the materials used to make the samples.

Brand	Manufacturer	Basic chemical composition
Reliner Kooliner GC	GC America Inc., Alsip, USA	Acrylic monomer, such as methyl methacrylate, and polymerization initiator.
Reliner TDV-Cold	TDV Dental Ltda., Pomerode, Brazil	Polymethacrylate. Pink powder: Polymethacrylate and pigment. Liquid: Methacrylates, plasticizer, initiator, and essence. Glaze: Polymethacrylate, ethyl acetate, and essence. Insulator: Liquid petroleum jelly.
Reliner Ufi Gel Hard C	VOCO GmbH, Cuxhaven, Germany	Methyl methacrylate-free, self-curing reline material with Neutral taste and odour
Adhesive Fixodent	Haleon, Brentford, UK	Silicon Dioxide, Sodium Carboxymethyl Cellulose, 40% Ca/20% Zn Gantrez Salt (Maleic Acid Polyvinyl Methyl Ether), Mineral Oil, Petrolatum Silica, Pigments Red 27 Lake
Adhesive Poligrip	Procter & Gamble (P&G), Cincinnati, USA	Silicon Dioxide, Sodium Carboxymethylcellulose, 40% Ca/20% Zn Gantrez Salt (Maleic Acid Polyvinyl Methylether), Mineral Oil, Petrolatum.
Adhesive Corega	Haleon, Brentford, UK	Partial sodium/calcium salt of poly (methyl vinyl ether/maleic acid), carboxymethylcellulose, mineral oil, petroleum jelly.

### Fabrication of the samples

The 120 samples were prepared in a matrix with dimensions of 22 mm (Ø) × 3 mm. Each reliner was handled according to the manufacturer’s instructions at 23°C ± 2°C with a relative humidity of 50% ± 10% [10]. Then, after the polymerization time and consistency indicated for use, each reliner was placed into the aforementioned metal matrix, and a pressure of 1.2 Kg with a hydraulic press was applied until its final polymerization. After separating the samples, they were polished with different grits of sandpaper (280, 320, 600, 1200, Norton, Sao Paulo, Brazil) for 3 min (sum of the times each sandpaper was used) and then polished with a felt disc and 1/4 micron diamond solution (Buehler, Lake Bluff, USA).

The specimens were polished with pumice stone and Spanish white (Vigodent, Rio de Janeiro, Brazil) for 3 min on each surface under water cooling. The specimens were polished using a polishing machine (APL-4, Arotec, Cotia, Brazil) [9]. After the final polishing, the first (initial – T0) reading was taken of the samples for the color change, microhardness, and roughness tests. In addition, EDS analysis and SEM were also carried out.

Subsequently, for each relining acrylic resin, there were four groups: group 1, no prosthesis adhesive was applied (control), group 2 applied Fixodent adhesive, group 3 applied Poligrip adhesive, and group 4 applied Corega adhesive. The control group for the three relining acrylic resins was sanitized daily with neutral soap and kept in an incubator in distilled water at 37°C for 2 months. The other samples were submitted to daily adhesive application. After sanitizing with neutral soap, the adhesives were applied again, and they were stored in incubators for 2 months until the color change, microhardness, and roughness evaluation methods were again carried out on the samples at T1 and T2.

### Color

Color readings were taken using an ultraviolet-visible reflectance spectrophotometer (UV- 2450, Shimadzu, Japan). The color change (ΔE) was calculated using the Commission Internationale de L’Eclairage (CIE) L*a*b* system, using the following formula: ΔE = [(ΔL) ^2^ + (Δa) ^2^ + (Δb) ^2^ ] ^½^ [11]. The ‘L’ represents the brightness from 0 (black) to 100 (perfect white), the ‘a’ represents the amount of red (positive values) or green (negative values), and the ‘b’ represents the amount of yellow (positive values) or blue (negative values) [10].

### Surface microhardness

Knoop microhardness was determined according to the American Society for Testing and Materials (ASTM) E384-11 [12] using a microhardness tester (HMV-2T; Shimadzu Corporation, Kyoto, Japan) with a load of 25 g for 10 s. For each sample, five measurements were taken, and all the values were later analyzed. Each sample was placed 500 μm from any other sample and 500 μm from the edge of the sample plates.

### Surface roughness (Ra and Rt)

Surface roughness was analyzed in accordance with ISO 21920 [13]. The analyses were carried out by means of contact profilometry, using an SJ-401 surface roughness profilometer (Mitutoyo, Kanagawa, Japan), featuring a 2 mm diameter diamond. The settings defined were: λ = 0.08 mm cutting wavelength and 0.25 mm transverse length at a speed of 0.05 mm/s for the surface roughness characteristics Ra and Rt. Ra is the average roughness, which is determined by the arithmetic mean of the absolute values of the ordinates of the roughness profile. Rt is the roughness depth, which represents the maximum peak height in the measured profile. Three measurements were taken on each sample, with the average defined as the roughness value [10].

The equation used to calculate the surface roughness was:

KHN = A/P

Where:

**A** = projected contact area

*A* = 0.070279 *d*^2^

Substituting A → gives the same final formula:


$$KHN=\frac{P}{0.070279\;d^{2}}$$

### Thermocycling

Samples were distributed according to time: T0 – initial analysis; T1 – stored in distilled water in an incubator (Scientific Equipment; Cienlab) at 37°C ± 2°C, and the adhesives were applied for 2 months; T2 – after the samples were submitted to thermocycling (Model MSCT-3, Convel). Thermocycling was performed in distilled water with alternating 30-second baths at temperature of 5°C ± 1°C and 55°C ± 1°C (70 s per cycle; dwell time: 30 s; transfer time: 5 s) in a total of 5.000 cycles. Thermocycling, under such conditions, represents a 6-month clinical aging of the acrylic resin [14, 15]. The evaluator was blinded regarding the type of resin analyzed.

### X-ray dispersive energy analysis and SEM

The topographic surface was evaluated qualitatively using SEM (JSM-5600LV, JEOL; Tokyo, Japan). Randomly selected samples (*n* = 1) from each group were coated with gold by cathodic concentration and tested in two areas under 1,000x and 500x magnifications. The chemical composition (atomic%) was evaluated by EDS in small volumes, on the order of 1 μm³, to identify the presence of C, O, Na, Al, Si, K, Zn, and Ba. The images obtained from the samples were tested at T0 and T2 for the elemental composition with energy-dispersive X-ray spectroscopy. Energy-dispersive X-ray spectroscopy spectra were obtained with an SEM (JSM-5600LV, JEOL; Tokyo, Japan) equipped with an SEM system. The primary electron energy was 15 keV. Other test stops included a 20 mm working distance, a 10-second process time, a 100-second active time, and a 30% to 40% final time [9, 16]. Both analyses were performed three-times in each sample [17].

### Statistical analysis

Statistical evaluations were carried out using Jamovi Software (Version 2.2, Jamovi Project, Australia). Data were assessed for normality using the Shapiro–Wilk test, and all data were shown to be normally distributed. Data on color, microhardness, and roughness were verified through repeated-measures analysis of variance (ANOVA) (time, resin, and adhesives as variables). When statistical differences were found, the Tukey HSD test was applied for post hoc comparisons, and, in all cases, the values were considered significant when *p* < 0.05.

## Results

The ANOVA of the color change values (ΔE) presented in Table 2 showed that all the factors – time, type of resin, and type of adhesive – had a statistically significant influence on color change. This indicates that the Kooliner GC, TDV-Cold, and Ufi Gel Hard C materials showed different levels of color change over time and as a function of the different adhesives used, as can be seen in Table 3. Although Ufi Gel Hard C maintained the lowest average ΔE values compared to the others, the data showed that all the factors tested contributed to significant variations in the color stability of the resins.

**Table 2:** Three-way ANOVA of value of color change (Tukey *P* < 0.05).

Factors	Sum of squares	df	Mean square	*F*	*p*
Time	1.238	1	1.2384	32.96	< 0.001
Time * Resin	0.308	2	0.1539	4.10	0.019
Time * Adhesive	3.673	3	1.2245	32.59	< 0.001
Time * Resin * Adhesive	0.282	6	0.0470	1.25	0.286
Residual	4.058	108	0.0376		

**Table 3:** Mean values and standard deviation on color change comparing resins, adhesives, and different times.

Resins	Adhesives	Delta 1	Delta 2	*P < 0.05
	Control	4.04 A,a ± 0.51	4.07 A,a ± 0.4	
TDV	Corega	3.9 B,a ± 0.34	4.04 A,a ± 0.47	
	Fixodent	4.05 A,a ± 0.29	4.05 A,a ± 0.32	
	Poligrip	4.09 A,a ± 0.4	4.05 A,a ± 0.41	
	Control	3.52 C,a ± 0.13	3.7 B,b ± 0.38	
Kooliner	Corega	3.52 C,a ± 0.28	3.7 B,b ± 0.44	
	Fixodent	4.02 AB,a ± 0.42	3.8 B,b ± 0.34	
	Poligrip	3.54 C,a ± 0.44	3.8 B,b ± 0.39	
	Control	3.34 D,a ± 0.11	3.8 B,b ± 0.3	
Ufi Gel Hard C	Corega	3.4 D,a ± 0.15	3.81 B,b ± 0.26	
	Fixodent	3.86 B,a ± 0.25	3.74 B,a ± 0.24	
	Poligrip	3.38 D,a ± 0.14	3.8 B,b ± 0.2	
*P < 0.05	*	*	*	*

Note: Averages followed by the same uppercase letter in the column and lowercase letter in the row do not differ at the 5% significance level (*P* < 0.05) using the Tukey test. Delta 1: color change between T0 and T1; Delta 2: Color change between T1 and T2.

The three-way ANOVA on surface microhardness (Knoop) shown in Table 4, demonstrated that the ‘Time’ factor had a statistically significant effect on the microhardness of the materials tested. There was also a significant interaction between the types of relining acrylic resin, indicating that the increase in microhardness over time was different between the materials. In addition, the analyses demonstrated that types of adhesives influence microhardness over time.

**Table 4:** Three-way ANOVA on surface microhardness values (Tukey *P* < 0.05).

Factors	Sum of squares	Df	Mean square	*F*	*p*
Time	499.53	2	249.767	568.967	< 0.001
Time * Resin	206.09	4	51.522	117.367	< 0.001
Time * Adhesive	6.71	6	1.118	2.547	0.021
Time * Resin * Adhesive	4.82	12	0.402	0.915	0.532
Residual	94.82	216	0.439		

The data in Table 5 demonstrates an increase in microhardness measurements of the materials. UFI Gel Hard C had the highest increase, Kooliner GC the lowest. Surface microhardness increased for all tested materials after thermocycling, as shown in the average values (Knoop) between T0 and T2. This increase was more evident for some groups, such as the Ufi Gel Hard C material.

**Table 5:** Mean values and standard deviation of the change in surface microhardness (Knoop) comparing resins, adhesives, and different times.

Resins	Adhesive	T0	T1	T2	*P < 0.05
	Control	7.7 A,a ± 0.22	8.58 A,b ± 0.6	9.56 A,c ± 0.64	
TDV	Corega	7.7 A,a ± 1	8.62 A,b ± 1	9.7 A,c ± 0.86	
	Fixodent	7.9 A,a ± 0.95	8.58 A,b ± 0.74	9.56 A,c ± 0.69	
	Poligrip	7.9 A,a ± 0.69	8.58 A,b ± 0.76	9.8 A,c ± 0.48	
	Control	8.5 B,a ± 0.65	8.9 B,b ± 0.54	9.8 B,c ± 0.53	
Kooliner	Corega	8.5 B,a ± 0.53	8.9 B,b ± 0.7	10 B,c ± 0.55	
	Fixodent	8.8 B,a ± 0.5	8.7 B,b ± 0.57	9.8 B,c ± 0.71	
	Poligrip	8.8 B,a ± 0.51	8.9 B,b ± 0.87	10.2 B,c ± 0.83	
	Control	16.6 C,a ± 1.22	20 C,b ± 0.69	22 C,c ± 0.75	
Ufi Gel Hard C	Corega	16.6 C,a ± 1.7	20.8 C,b ± 1.30	22.6 C,c ± 0.92	
	Fixodent	16.9 C,a ± 1.07	20 C,b ± 0.7	22 C,c ± 0.89	
	Poligrip	16.9 C,a ± 1.11	20 C,b ± 0.7	22.8 C,c ± 0.76	
*P < 0.05	*	*	*	*	

Note: Averages followed by the same uppercase letter in the column and lowercase letter in the row do not differ at the 5% significance level (*P* < 0.05) using the Tukey test.

Table 5 also demonstrates that all the samples showed a significant increase in values over time (T0, T1 and T2), regardless of the adhesive used (Control, Corega, Fixodent, or Poligrip). On the other hand, the average microhardness values at each time showed no significant differences between the adhesives. These results suggest that the time factor, possibly associated with the action of thermocycling, had a greater influence on the increase in microhardness than the type of adhesive applied.

**Figure 2:**
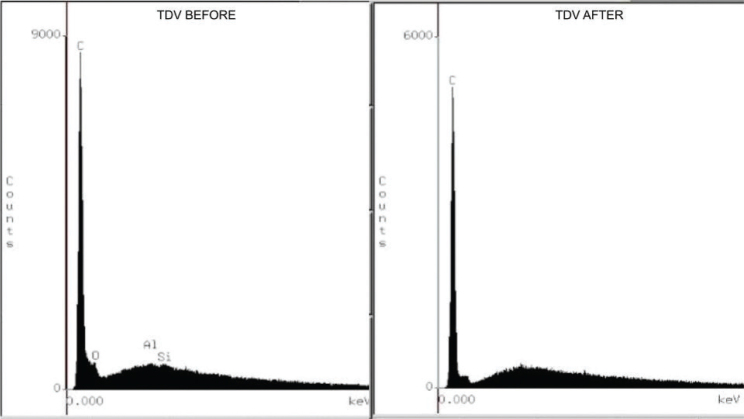
Energy dispersive X-ray spectroscopy (EDS), before and after TDV-Cold relining acrylic resin resurfacing.

**Figure 3:**
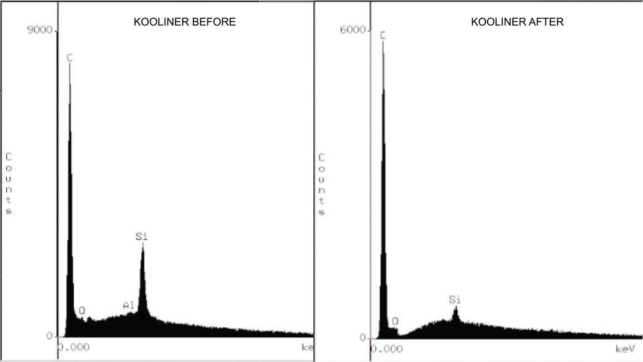
Energy dispersive X-ray spectroscopy (EDS), before and after Kooliner GC relining acrylic resin resurfacing.

**Figure 4:**
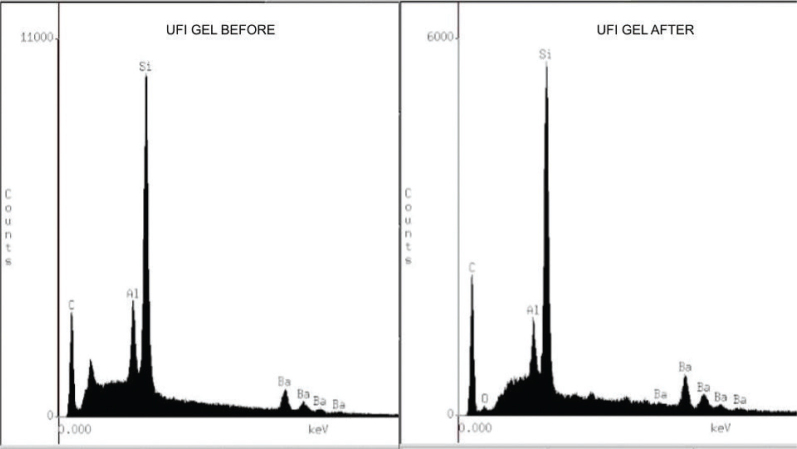
Energy dispersive X-ray spectroscopy (EDS), before and after Ufi Gel Hard C relining acrylic resin.

Analysis by energy dispersive X-ray spectroscopy (EDS) revealed changes in the chemical composition of the resin’s surface. In the initial reading, the Ufi Gel Hard C resin demonstrated greater compositional complexity, with the presence of silicon (Si), carbon (C), aluminum (Al), and barium (Ba), suggesting a greater number of inorganic fillers. Kooliner had C, Si, and traces of Al, while TDV contained C, Al, Si, and oxygen (O). After applying the adhesives and thermocycling, there was a general reduction in the concentration of inorganic elements in all the materials (Figures 2–4), and the application of the adhesives did not interfere with the elements present.

In the energy dispersive X-ray spectroscopy (EDS) analysis, all the fillers showed a reduction in the concentration of elements in their composition after thermocycling. The composition of Ufi Gel Hard C showed higher concentrations of the elements Silicon and Barium compared to the other relining acrylic resins, even after thermocycling for 5.000 cycles.

The ANOVA for roughness shown in Table 6 indicated that the time had a statistically significant effect on the surface roughness of the materials analyzed. Table 7 shows an increase in roughness between times T0 (initial), T1 (post-adhesive), and T2 (after thermocycling) for the TDV-Cold, Kooliner GC, and Ufi Gel Hard C materials. Table 5 shows that the TDV-Cold material had the lowest initial value (T0 = 0.118 µm), but its roughness increased progressively until T2 (0.200 µm). The Kooliner GC resin always showed intermediate values, but also increased. Ufi Gel Hard C, on the other hand, had the highest initial values and maintained the highest roughness over time, although with a more discreet increase between times (0.183 → 0.188 → 0.205 µm).

**Table 6:** Three-way ANOVA of roughness change values (Tukey *P* < 0.05).

Factors	Sum of squares	*df*	Mean square	*F*	*p*
Time	0.24503	2	0.1225	417.09	< 0.001
Time * Resin	0.05953	4	0.0149	50.66	< 0.001
Time * Adhesive	0.00213	6	3.54e-4	1.21	0.304
Time * Resin * Adhesive	0.00425	12	3.55e-4	1.21	0.280
Residual	0.06286	214	2.94e-4		

**Table 7:** Mean values and standard deviation of roughness change.

Resin / time	T0	T1	T2	*P < 0.05
TDV	0.118 Aa ± 0.01	0.147 Ab ± 0.02	0.2 Ac ± 0.02	*
Kooliner	0.131 Ba ± 0.01	0.182 Bb ± 0.01	0.21 Ac ± 0.01	*
Ufi Gel Hard C	0.183 Ca ± 0.02	0.188 Cb ± 0.01	0.205 Ac ± 0.02	*
*P < 0.05	*	*	*	

Note: Averages followed by the same uppercase letter in the column and lowercase letter in the row do not differ at the 5% significance level (*P* < 0.05) using the Tukey test.

When analyzing Tables 6 and 7, over the times evaluated (T0, T1, and T2), it was observed that the roughness varied between the different materials, indicating a significant interaction between time and type of relining acrylic resin. When comparing the materials, statistically significant differences were found at times T0 and T1, suggesting that the type of relining acrylic resin influenced the behavior of the surface in the early stages. However, at T2 (after thermocycling), the roughness values between the resins were close, suggesting a tendency for the surface characteristics to level out after simulated aging for 5.000 cycles. The interactions related to the adhesives were not statistically significant, indicating that the application of the different adhesives had no direct impact on the surface roughness of the relining acrylic resins over the periods.

**Figure 5:**
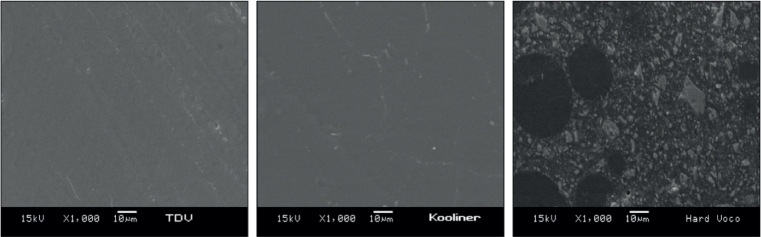
Scanning electron microscope (SEM) of the three relining resins, TDV, Kooliner and Ufi Gel Hard C.

Figure 5 obtained by SEM showed differences in the surface of the relining acrylic resins analyzed, specifically Ufi Gel Hard C, which had a more irregular and heterogeneous morphology. In contrast, the surfaces of the TDV-Cold and Kooliner GC resins were smoother and more homogeneous, with fewer apparent irregularities. These visual findings are in line with the roughness analysis data, which indicated higher values for Ufi Gel Hard C, while TDV and Kooliner showed lower and similar values.

## Discussion

The null hypothesis that the adhesives would not influence color change, microhardness, and roughness of the relining acrylic resins before and after aging by thermocycling was rejected since several changes could be observed throughout the analyses.

Regarding color, according to the studies by Goldstein and Schmitt [18] we consider that ΔE is acceptable within the clinically acceptable limit of 3.7. However, there is controversy over this value, as other studies suggest that the clinically acceptable limit is 3.3 [16]. According to Johnston and Kao, ΔE values greater than 3.3 indicate a clinically visual perception of color change [19]. Douglas et al. [20] reported that a ΔE value above 2.6 is clinically perceptible, and greater than 5.5 is clinically unacceptable. The lower the average color change values (ΔEab), the greater the color stability of the material [21]. In this context, the color change is noticeable in all relining acrylic resins but within clinically acceptable parameters, as shown in Table 3. This is an important factor, as the aesthetics of prostheses are fundamental to patient satisfaction.

Color change in acrylic resins can be increased by exposure to staining agents, which reinforces the need to choose the materials used for removable prosthesis bases with caution [6]. The interaction between materials and adhesives is another critical factor that can influence color stability. In this study, statistical analysis indicated significant differences in color change depending on the type of adhesive. This can be explained by the fact that the Fixodent adhesive contains a dye called Cl 45410 (Red 27 Lake). Its structure (bromine/chlorine and phenolic groups) favors interactions with the organic matrix of the adhesive and photochemical reaction, therefore it tends to exert a greater influence on the final color of the product than inert pigments [22].

The study simulated a clinical condition in which many other factors can affect color. It is important to emphasize that other factors, isolated or associated, such as poor hygiene of the prosthesis, components, particles from the oral environment, porosity of the material associated with the manufacturing technique, flaws in the surface of the material, and the polishing surface can impact on the color stability of acrylic resins [23].

Roughness, for example, can allow the infiltration of coloring substances, while an inadequate surface finish can facilitate the accumulation of particles that alter the color [23]. Considering the results of these studies, the final roughness was similar between the three different resins. However, Ufi Gel Hard C had a lower ΔE in the color reading compared to TDV-Cold and Kooliner GC, so the color change was not related to roughness.

Thermocycling caused significant color changes in the resin samples. Studies have shown that humidity and heat are factors that can cause polymer degradation [24, 25]. Higher temperatures can also cause gas porosity when boiling the monomer. The biomechanical and aesthetic properties of acrylic resins have been well proven, although several aspects still need to be improved, such as polymerization characteristics, dimensional changes due to water absorption and antibacterial properties [26].

The variation between brands in terms of susceptibility to color change can be explained by the product’s different composition. TDV-Cold’s more obvious color change may be due to its molecular structure [9]. Studies have shown that water absorption is the main cause of color change in resins [11]. Therefore, TDV-Cold may have a structure that influences greater absorption.

Another factor that may explain the difference in color between the resins and its alteration is the presence of Ba (Figures 2–4). Thermocycling promotes a reduction in the intrinsic elements of the resins’ composition, indicating possible leaching of components or surface degradation related to the action of water or aging. This loss can affect optical properties, especially color stability. Ba acts as a color stabilizer and opacifier and reduces degradation due to UV radiation and humidity [10, 26]. The consistent presence of Ba in the composition of Ufi Gel Hard C resin, even after thermocycling, contributed to its greater color stability over time. This inorganic particle acts as a structural filler and optical stabilizer, making the resin less susceptible to surface alterations [10, 27, 28].

Regarding surface microhardness, Knoop microhardness test was performed because the reliners were acrylic hard, which requires this type of analysis [29]. The results showed that the materials differed in hardness and that it increased over time, as shown in Table 3. Neppelenbroek et al. [30] reported a continuous increase in the surface microhardness values of the acrylic resins analyzed up to 60 days of storage in water, explaining that the release of residual monomer from the resin bases contributed to an increase in surface microhardness after storage in water [23–25] and thermocycling. The increase in hardness after thermocycling may be related to the complete polymerization of the liner and to the change in temperature, which can cause the material to shrink [24, 25, 31, 32]. Studies have shown that exposure to heat during accelerated aging, thermocycling, probably favored the evaporation of water present in the resins, contributing to the removal of residual monomer from the surface. This process may be related to the observed increase in surface microhardness values, since the elimination of residual monomer is often associated with an improvement in this mechanical property [33–35].

The decrease in inorganic elements, especially Si, may have an impact on properties such as microhardness, which may explain the observed mechanical results. The greater presence of these elements in the composition of Ufi Gel Hard C resin explains its higher microhardness value, since these particles act as fillers [36, 37]. The microhardness results showed higher surface microhardness in the Ufi Gel Hard relining acrylic resin at all reading times, which is related to the significant presence of silica in its composition, as analyzed by EDS and SEM images. The presence of silica is not observed in the composition of the TDV resin. Higher surface microhardness values can be interpreted as positive, since the higher the hardness, the lower the possibility of scratches and possible fractures in the material [24, 25, 32].

Studies have reported that the median microhardness of Kooliner GC can vary between 7.0 ± 3.44 KHN [29], while others have reported that it can vary between 9.09 ± 1.61 [38] (Table 5). Taking these values into account, the values obtained in this study are within the acceptable range. No specific Knoop microhardness values were found in the literature for TDV-Cold and Ufi Gel Hard C relining acrylic resins. However, studies using the Vickers scale to compare materials indicate that TDV-Cold resin tends to have lower microhardness values, while Ufi Gel Hard C consistently shows higher values than the other luting resins evaluated, in agreement with this study [38].

It is likely that during storage and thermocycling, water molecules penetrate the areas between the polymer chains and push them further apart. Secondary chemical bonding forces (van der Waals forces) decrease between the polymer chains. Thus, water molecules can act as plasticizers that damage the mechanical strength of the material through the formation of microcracks related to the absorption and hydrolytic degradation of the polymer, resulting in the cleavage of the bond and the gradual deterioration of its infrastructure influences the microhardness of TDV and Kooliner resins [38]. The increase in microhardness after accelerated aging was likely the result of complete polymerization of the resin that occurred when the samples were subjected to different types of energy (high temperature through thermocycling, storage at 37°C for 2 months) (Table 5). After the aging periods, the Barium groups exhibited significantly higher microhardness values compared to the other groups. Similar to the color change results, this result can be explained by the presence of BaSO4 (Figures 2–4), which would promote a strong bond to the resin particles and prevent their degradation [39].

Concerning roughness, Tables 6 and 7 showed that it increased in different relining acrylic resins over time. Analysis of surface roughness showed that Ufi Gel Hard C relining acrylic resin had the highest initial values among the materials evaluated, due to the presence of silica (Table 7 and Figures 2–4). This difference was also confirmed by SEM, in which the surface of Ufi Gel was visually more irregular than the others. This roughness may be related to its more complex composition and the presence of a greater quantity of inorganic particles, as evidenced by EDS and the significant presence of silica in its composition. A more irregular surface can have an impact on aesthetics and facilitate microbial adhesion, despite contributing to mechanical resistance [40, 41]. Therefore, the lower roughness of TDV-Cold and Kooliner GC resins suggests better surface smoothness, which can favor patient comfort and hinder the accumulation of biofilm, important characteristics for the clinical success of prostheses.

The increase in the roughness of the resins may be related to the possible loss of soluble components, such as plasticizers, leaving empty spaces. These empty spaces are responsible for the roughness and the increase in size, resulting in porosities, which may increase with the handling of the materials used in cleaning and adhesives. Thermocycling, combined with cleaners and adhesives, causes deterioration [42]. Changes in surface roughness have a direct impact on microbial adhesion, as the irregularities present act as refuges for microorganisms, favoring their permanence on surfaces even after usual hygiene procedures [43, 44]. Thus, controlling surface roughness is essential to ensure the clinical performance of materials, since it is related to extrinsic pigmentation, color change, adhesion of microorganisms, oral tissue health, and patient comfort [44]. The physicochemical changes that affect color result from internal reactions that are mainly caused by the initiator system, the type of monomers, the breaking of polymer chains by UV light, oxygen crosslinking, leaching of plasticizers, monomers or pigments, water absorption, dye changes, and surface roughness [44].

Although the statistics revealed no significant differences in the changes in microhardness and roughness, and the color change was clinically acceptable, the type of adhesive used remains an important consideration. This is because different adhesives can have different properties, which, although they did not manifest a significant difference in the change in roughness and microhardness in this study, can influence the overall performance of the prosthesis in varying clinical conditions.

In addition to the in vitro study, clinicians should be involved to confirm the changes made to the hard reliner in the intraoral environment. The need for specific studies on the physical, mechanical, and optical properties of hard reliners over time highlights the need for further research. Future studies with a larger sample size for the EDS and SEM analyses can also confirm the results described.

## Conclusion

The relining acrylic resins analyzed showed distinct behavior in response to aging by thermocycling, with Ufi Gel Hard C standing out for its greater stability. Time was the main factor influencing color, microhardness, and roughness properties

## Conflicts of interest

The author(s) declared no potential conflicts of interest
